# Understanding the factors influencing biosecurity adoption on smallholder poultry farms in Ghana: a qualitative analysis using the COM-B model and Theoretical Domains Framework

**DOI:** 10.3389/fvets.2024.1324233

**Published:** 2024-07-23

**Authors:** Anica Buckel, Kofi Afakye, Eric Koka, Cortney Price, Emmanuel Kabali, Mark A. Caudell

**Affiliations:** ^1^Animal Production and Health Division/Joint Centre for Zoonotic Diseases and Antimicrobial Resistance, Food and Agriculture Organization of the United Nations, Rome, Italy; ^2^Emergency Centre for Transboundary Animal Diseases, Food and Agriculture Organization of the United Nations, Nairobi, Kenya; ^3^Regional Office, Food and Agriculture Organization of the United Nations, Accra, Ghana; ^4^Department of Sociology and Anthropology, University of Cape Coast, Cape Coast, Ghana; ^5^Office of Innovation, Food and Agriculture Organization of the United Nations, Rome, Italy

**Keywords:** behavioral science, AMR, biosecurity uptake, Theoretical Domains Framework (TDF), COM-B, knowledge–action gap, poultry, Ghana

## Abstract

**Introduction:**

Antimicrobial resistance (AMR) poses a significant global threat to public, animal, and environmental health, consequently producing downstream economic impacts. While top-down approaches to addressing AMR (e.g., laws regulating antimicrobial use) are common in high-income countries, limited enforcement capacities in low- and middle-income countries highlight the need for more bottom-up approaches. Within agriculture, efforts to apply bottom-up approaches to AMR have often focused on the promotion of biosecurity, which should reduce the need for antimicrobials by mitigating disease risk and limiting AMR transmission. Traditionally, efforts to encourage biosecurity adoption have emphasized training and awareness-raising initiatives. However, a growing body of research suggests a disconnect between knowledge and behavior, highlighting the existence of a knowledge–action gap.

**Method:**

To understand the barriers and enablers patterning the knowledge-action gap in on-farm biosecurity uptake, we draw upon models from behavioral science. We analyzed in-depth interviews and two focus group discussions with smallholder poultry producers in Ghana to understand factors underlying the intention–action gap in adopting biosecurity. As an analytical framework, we draw upon the Theoretical Domains Framework in combination with the Capability-Opportunity-Motivation Behavioral Model.

**Results and discussion:**

While smallholder poultry farmers in Ghana were aware of the importance of biosecurity practices, they struggled with consistent implementation. Financial constraints, challenges in adapting practices to the local context, and limited resources hindered adoption. Additionally, cognitive biases like prioritizing short-term gains and underestimating disease risks played a role. However, some farmers found motivation in professional identity and social influences. These findings highlight the need for designing biosecurity interventions that consider human behavioral factors and the context in which behavior occurs. This underscores the importance of collaboration across disciplines, including veterinary science and the social and behavioral sciences. Implications and recommendations for researchers and practitioners are discussed.

## 1 Introduction

Increasing rates of antimicrobial resistance (AMR), when microorganisms such as bacteria and viruses survive treatment from antimicrobial drugs thereby rendering these drugs less effective, impact public, animal, and plant health and produce downstream impacts on socio-economic development. A systematic analysis of global mortality estimated that, in 2019, ~1.27 million individuals succumbed to resistant bacterial infections (1). By 2030, the World Bank estimates the economic impact of AMR will be over 1 trillion USD annually, through impacts on health costs, labor availability, trade and on agricultural production (2). AMR's associated risks are closely tied to its presence in plants and animals by jeopardizing food security, agricultural output, and can directly impact public health through the spread of AMR through human-animal interactions and foodborne transmission. Moreover, as about 70 percent of antibiotics used in livestock are medically relevant for humans, the misuse of antimicrobials in agriculture can erode their effectiveness in public health (3).

The impact of AMR within agri-food systems has spurred concerted intervention efforts by governments, non-governmental actors, and intergovernmental agencies. Efforts are often guided by national and global action plans, such as the Food and Agricultural Organization Action Plan on AMR 2021–2025 (4). These plans usually include the objectives of raising awareness, strengthening surveillance, enabling good production practices, promoting the responsible use of antimicrobials, and strengthening governance. Of these objectives, considerable resources have been devoted to promoting the responsible use of antimicrobials, as misuse of antimicrobials is considered a main driver of AMR (5). Often, these efforts involve top-down governance mechanisms meant to regulate and control the supply and use of antimicrobials in agriculture. In 2022, for example, the European Union banned the routine use of antibiotics in agriculture with antibiotics only to be used for treatment of individual animals (6).

However, in many low- and middle-income countries (LMICs), the efficacy of top-down regulations on antimicrobials is challenged by limited governance capacities and characteristics of agri-food systems. Although many LMICs have antimicrobial regulations targeted at the agricultural sector (e.g., prescription requirements), underfunded national drug regulatory agencies hinder enforcement (7). Moreover, a shortage of veterinary professionals in LMICs makes implementing regulations at the farm level difficult. For example, a study in five African countries found that the ratio of veterinarians to livestock is about 20 times lower than that of high-income countries such as Denmark, France, Spain, and the USA (8). Finally, production systems in most LMICs are dominated by small-scale producers (9), adding complexity to regulatory oversight compared to high-income countries where comparatively fewer large-scale operations usually drive production.

The challenges associated with top-down approaches emphasize the need for bottom-up approaches to address AMR within agriculture in LMICs, including those that promote good practices such as biosecurity. Biosecurity, defined as all measures to prevent the introduction of pathogens to the farm and reduce the spread of pathogens on the farm, can impact both the emergence and spread of AMR. By limiting the emergence of disease, biosecurity can reduce the use of antimicrobials within livestock and, by limiting the spread of pathogens, can curb the transmission of AMR within and outside the farm (10). Consequently, interventions that indirectly address AMR through biosecurity are gaining importance (11), especially in contexts where top-down approaches are less effective. Importantly, however, there seems to be general agreement in the literature that the adoption of biosecurity on small-scale farms is often limited, with a disconnection between industry-recommended biosecurity standards and livestock producers' practices across countries (12, 13).

Considering the limited uptake of recommended biosecurity practices, there has been increased interest among researchers in understanding producers' actions in terms of the behavioral drivers and barriers related to disease control and prevention. This is evident by the increased employment of socio-psychological theoretical frameworks in the body of research in the field of veterinary epidemiology (14). However, there is a lack of application of empirically validated theoretical frameworks to better understand the determinants of producers' behavior and to design more effective behavior change interventions. Instead, biosecurity studies have been focused on understanding knowledge, attitudes, and intentions, as well as personal influences and relationships between producers and veterinarians (15, 16).

Notably, the intention to implement biosecurity measures and knowledge of the benefits of such measures are often poor predictors of actual adoption. This discrepancy between intentions and actions, known as the “knowledge–action gap”, has been consistently observed in Knowledge, Attitude, and Practice (KAP) studies across a variety of countries and livestock production contexts, including poultry in Ghana (17); pigs in the UK (18); pigs, poultry, and fish in Vietnam (19), and Maasai pastoralists in Tanzania (20, 21) and received recent attention in discussions about biosecurity adoption (22). Progress in addressing the knowledge-action gap has been hindered by several factors, including the tendency of humans to explain one another's behaviors through a folk psychological lens, which assumes rationality (23–25). This rationality assumption, however, assumes that individuals possess perfect information, a flawless understanding of their goals, and the ability to utilize this information to make decisions that align with their objectives (26). Finally, one of the most tangible barriers to progress may be the abundance of behavioral models to choose from when attempting to understand behaviors. Even when one or more models or theories are chosen, they do not cover the full range of possible influences, so they exclude potentially important variables (27).

In response, Michie and colleagues developed the Theoretical Domains Framework (TDF) and the Capability-Opportunity-Motivation Behavioral (COM-B) Model to help practitioners better understand behavior and design more effective behavior change interventions. The TDF consolidated 33 models of behavior or behavior change and includes 128 separate constructs and organizes them into 14 key domains, each representing distinct factors that influence behavior (see Table 1) (28, 29). In contrast, the COM-B Model was developed to make drivers of behavior more accessible by only focusing on the minimal factors necessary to drive behavior change: capability, opportunity, and motivation. The authors define capability as the individual's psychological and physical capacity to engage in the activity concerned. It includes having the necessary knowledge and skills. Motivation is defined as all those brain processes that energize and direct behavior, not just goals and conscious decision-making. It includes habitual processes, emotional responding, as well as analytical decision-making. Opportunity is defined as all the factors outside the individual that make the behavior possible or prompt it. Within these three components of the COM-B Model, additional subdivisions capture essential distinctions from the research literature. For instance, capability is divided into physical and psychological capability, while opportunity is divided into physical and social opportunity. Motivation is distinguished between reflective and automatic processes (27).

**Table 1:** Mapping of the behavior change wheel's COM-B system to the TDF domains (28).

**COM-B component**		**TDF domain**
Capability	Psychological	Knowledge
		Skills
		Memory, Attention and Decision Processes
		Behavioral Regulation
	Physical	Skills
Opportunity	Social	Social Influences
	Physical	Environmental Context and Resources
Motivation	Reflective	Social/Professional Role & Identity
		Beliefs about Capabilities
		Optimism
		Beliefs about Consequences
		Intentions
		Goals
	Automatic	Social/Professional Role & Identity
		Optimism
		Reinforcement
		Emotion

While the TDF and COM-B frameworks have seen extensive use in various fields, application of these frameworks in agriculture and in the context of AMR has been limited, with notable exceptions primarily focusing on antimicrobial use (30–33). Here, we draw upon the TDF and COM-B frameworks to analyze in-depth interviews with small-scale poultry producers in Ghana, focusing on understanding the barriers and enablers of good biosecurity practices. Reflexive thematic and framework analysis is used to identify the main barriers and enablers of good biosecurity practices. This analysis discusses the benefits and challenges of applying the TDF and COM-B frameworks in understanding the intention–action gap in biosecurity and as tools to address AMR, and recommendations are provided.

## 2 Materials and methods

### 2.1 Study context

This study was part of a broader research project initiated in 2019 by the Food and Agriculture Organization (FAO) in collaboration with the Ghanaian Government and the University of Cape Coast. The project focuses on addressing AMR within Ghanaian poultry systems. The research commenced with KAP assessments involving 109-layer poultry producers, which later led to the implementation of Farmer Field Schools (FFS). FFS is an agriculture extension approach where groups of 20–30 producers meet regularly on a host farm within a community, typically spanning an entire production cycle. During these meetings, producers engage in good production practices, with a strong emphasis on biosecurity, and conduct experiments to find solutions to production challenges. One layer FFS program lasting 32 weeks was conducted, with a total of 30 participants completing the courses [see Caudell et al. (34) for more details and results].

### 2.2 Study location and sampling

Qualitative interviews were conducted with poultry producers in Kade (pop. ≈ 20,000), the capital city of Kwaebibirem Municipality in the Eastern Region of south Ghana. Within the municipality, the predominant occupation is agriculture, which engages 76.8% of the economically active labor force. Major crops cultivated are oil palm, citrus, cocoa, maize, plantain, and cassava; livestock include ruminants and pigs, with some farmers engaging in aquaculture. Both layer and broiler birds are kept within the district, with an estimated 60 registered poultry farms and an average poultry farm size of 1,000 birds. More broadly, there were an estimated 2,227,817 layer birds within the Eastern Region, which places the Eastern Region as the fourth largest layer-producing region out of the 10 regions in Ghana. However, some caution should be exercised with these estimates as the last estimates are from 2009 (35). Prevalent poultry diseases in the municipality are Newcastle, Gumboro, and fowl pox. Within the municipality, as in many parts of Ghana, broiler keeping is usually a seasonal activity, with broiler batches kept meeting demand during the Christmas and Easter holidays.

Fifteen individual interviews (IDIs) and two focus group discussions (FGDs) were conducted with the same participants. To prevent potential response contamination, IDIs preceded the FGDs. Participants were purposely drawn from the pool of FFS attendees in Kade. These attendees were recruited for participation in the FFS by first approaching producers who had provided data during the KAP surveys. The selection was then expanded through word of mouth until between 30 and 35 producers agreed to participate for each school [for more details, see Caudell et al. (34)].

Working with FFS participants for a qualitative approach offers several advantages in understanding the intention–action gap. Firstly, FFS attendees have an established rapport with the researchers and facilitators, fostering a comfortable and trusting environment for open discussions and reducing social desirability bias. Secondly, FFS participants tend to possess higher levels of knowledge and awareness regarding biosecurity and AMR. This helps to identify and understand knowledge–action gaps by tapping into participants' potential barriers to translating knowledge into action.

### 2.3 Study procedure

IDIs and FGDs followed a semi-structured guide. The topics covered included animal health-seeking behaviors, biosecurity practices, farm management, awareness levels, and barriers or facilitators to adherence. Duration of the IDIs ranged from 20 to 30 min, while the FGDs were conducted in 60–80 min. To assess participants' awareness of biosecurity and identify barriers and facilitators to adoption, successive free listing was employed. Participants initially listed known biosecurity and then generated additional lists focusing on barriers and facilitators related to specific biosecurity measures mentioned. Interviews were conducted in English, with a translator available if participants preferred to speak Twi. All interviews were audio-recorded to enhance accuracy and enable thematic analysis.

### 2.4 Data analysis

Audio-recorded semi-structured interviews were transcribed verbatim and, when necessary, translated into English. Transcriptions were then anonymized before data were analyzed independently by the two authors, AB and MC, to reduce biases and subjectivity. The free and open-source qualitative research tool *Taguette* was used for data analysis (36).

Initially, inductive codes were generated through a reflexive thematic analysis (37). This process involved examining the transcripts to identify emerging themes and codes without predetermined categories. Subsequently, the transcripts were revisited to generate deductive codes. The Theoretical Domains Framework (TDF) served as the analytical framework in this step. Each TDF domain represented a code (e.g., “Belief About Consequences”). Both authors compared and discussed the codes generated independently before reaching a consensus. Finally, the identified inductive and deductive codes were mapped onto the COM-B model.

### 2.5 Ethical consideration

The informed consent process for this study emphasized participants' agency. Enumerators explained the study's aims and objectives, including the benefits and risks of participation, allowed participants to ask questions before obtaining their consent, and clarified their right to withdraw from the study at any time. Participants were assured of confidentiality and anonymity in data usage. Verbal consent, along with separate consent for audio recording, was obtained. Permission to conduct the study was approved by the Ministry of Health Ethical Review Board (ID No. 014/10/19).

Confidentiality was maintained by de-linking personal identifiers from the collected data and assigning unique participant identifiers. Data were stored securely on password-protected devices accessible only to the study team. Audio recording of FGDs and IDIs was conducted with participants' consent, offering the option to pause or suspend recording.

## 3 Findings

### 3.1 Participant information

The study sample comprised 15 poultry producers from the Denkyembour and Kwaebibirem Districts in Kade, Ghana. The mean age within the sample was 45 years (range = 31–64, SD = 10.6). Most participants were male (*n* = 14), with one female participant (*n* = 1). Layer flock sizes ranged from 500 to 4,000, although most producers reported smaller flock sizes (median = 867 birds, IQR = 700). Similarly, the years of poultry farming experience varied from 5 years to 40 years, but the distribution across respondents in experience was skewed toward fewer years (median = 15, IQR 13).

### 3.2 Qualitative results

**Figure 1:**
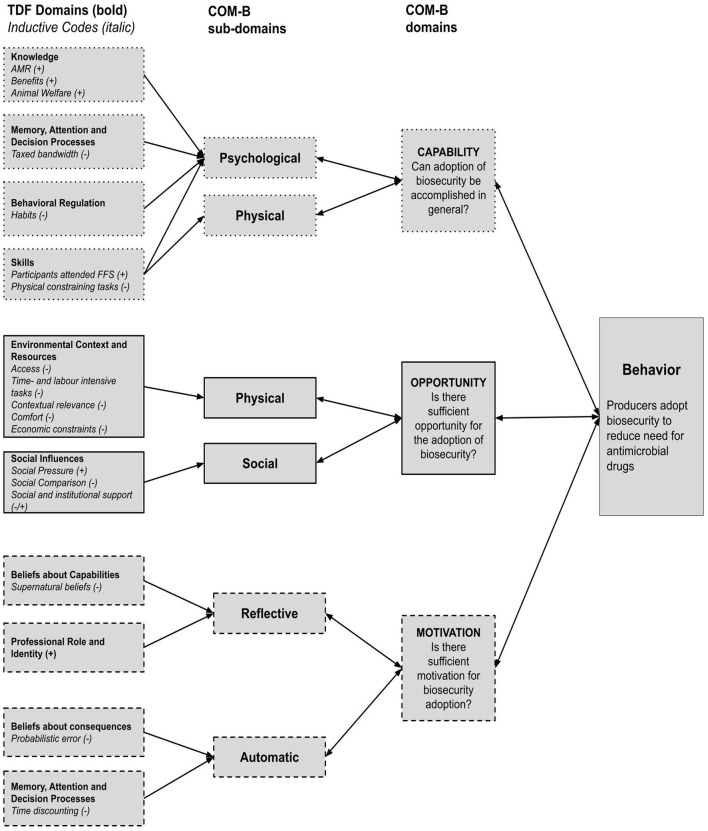
Combined COM-B & TDF analysis of factors on producers' biosecurity behavior. [Barriers (–) and Enablers (+)]. TDF domains are in bold with identified inductive themes in cursive.

Figure 1 below summarizes the identified inductive themes (cursive) and how they were grouped into the TDF and COM-B framework. In this section, COM-B components are first presented, and TDF domains are then discussed under the corresponding COM-B component according to Cane (28), with two exceptions: The TDF domain “Memory, Attention, and Decision Processes” and “Beliefs about Consequences” were both grouped under “Reflective Motivation”.

#### 3.2.1 Capability

Capability encompasses producers' psychological and physical ability to engage in the adoption of biosecurity.

##### 3.2.1.1 TDF: knowledge (an awareness of the existence of something)

Given participation in the FFS, the study sample was well-informed about biosecurity, why these practices should be implemented, and how they relate to AMR. One participant expressed this knowledge, stating, “*When doing the spraying and disinfection it prevents pathogens that are disease-causing organisms from causing cross-contact with other animals. [...] The spraying can deactivate those organisms.”* (FGD 1). Referencing links to AMR, one participant explained “*Indirectly when [eggs are] sold you are feeding people with antibiotics, which is not good.”* (Participant 3). At the same time, evidence of the knowledge–action gap existed in responses, with participants admitting that they were aware of practices to which they did not commit. “*I do not use the footbath though, but I have it.”* (Participant 3) and “*We know we are supposed to do it, but we don't do it because I'm saying we are not used to that.”* (FGD 1).

Participants in the study frequently also emphasized the importance of animal welfare, drawing parallels between the needs of their chickens and human needs. This concern for animal welfare was expressed in statements such as, “*Even as a human, you wouldn't like to fall sick, so in the same way, you have to ensure the birds do not also fall sick*” (Participant 6) and “*The birds are animals, alright, but they are also like people. I will give an example: when the litter is dirty and it is cleaned, you realize that a day after the litter is cleaned, the birds become happy and lively*” (Participant 4).

##### 3.2.1.2 TDF: behavioral regulation (anything aimed at managing or changing objectively observed or measured actions)

Habits emerged as a recurrent theme in FGDs and IDIs as a barrier to the consistent implementation of biosecurity practices. Participants reported that they were aware of and understood the importance of biosecurity practices, but that they often struggled to implement them consistently due to old habits. For example, one participant said, “*We know these hygienic practices. We have mentioned some that we do them regularly. But we have also brought them here again because we think that they are difficult for us to do regularly*.” (FGD 1). Another participant added, “*Even changing our clothing [is difficult].”*

Despite the challenges, some participants reported that they were able to overcome old habits and implement biosecurity practices consistently by building them into their daily routines. For example, one participant said, “*Washing of drinkers is routine and I do it every day*” (Participant 9). Another participant explained: “*It's easy for you to do it. Yes. So, you do it without thinking about it, every day*.” (FGD 2)

##### 3.2.1.3 TDF: skills (an ability or proficiency acquired through practice)

Practical concerns related to their physical ability to carry out certain biosecurity measures independently were raised among some participants. A common target of these concerns was litter management. Due to the physical challenges involved, participants may depend on others, such as their children or additional help, to assist them with tasks like litter management. When these helpers were unavailable, it created difficulties for the participants in completing these tasks. “*The most difficult to do now is the litter management. How to remove the litter is a problem. I do not forget to do it, but it is difficult, if my children are not available, I have to ask someone to assist me and that is [a] problem.”* (Participant 7). Some participants mentioned that they have a multitude of tasks to manage on their farms. In addition to litter management, there were other responsibilities, such as mixing feed.”*[I] mix my own feed so if I add changing litters [as well] it will be tedious, so I employ someone to do it for me*.” (Participant 13).

##### 3.2.1.4 TDF: memory, attention and decision processes (the ability to retain information, focus selectively on aspects of the environment and choose between two or more alternatives)

Participants in the study frequently mentioned feeling overwhelmed by the numerous tasks they must remember and perform in their day-to-day work. This sense of being overwhelmed was attributed not only to the volume of tasks but also to the complexity of some tasks. For example, one participant acknowledged, “*I follow (biosecurity practices) but as humans once a while you may not be able to keep charts.”* (Participant 8). This sentiment was shared by another participant who explained, “*Most farmers do everything on their own, removing eggs, changing feed, so most of them have a divided attention when we come.”* (Participant 10). In response to this sense of overwhelm, some participants mentioned attempting to simplify certain processes to make them more manageable. For instance, one participant shared, “*Me for instance, apart from changing my footwear all the time [before entering the pen], I put water in a bottle and sprinkle it in front of the pen and step into it when I want to enter the pen.”* (FGD 2).

Another participant explained that they reported trying to “dodge” the small costs that occur when regularly buying disinfectant, even though they were fully aware that the costs for treating their animals exceed prevention measures “*Preventing is lesser than managing a disease. It's human nature. We like dodging the small expenses [and] then meeting the higher expenses.”* (FGD 2).

#### 3.2.2 Opportunity

Opportunity reflects external factors influencing producers' willingness or capacity to adopt biosecurity. Opportunity can be physical, based on environmental contexts and resources, or social, based on social interactions and influences.

##### 3.2.2.1 TDF: environmental context and resources (any circumstance of a person's situation or environment that discourages or encourages the development of skills and abilities, independence, social competence, and adaptive behavior)

Several respondents indicated that access to products and services impacted participants' abilities to engage in biosecurity. Concerns were raised about access to veterinary services “*The truth of the matter is that in [location removed] there is only one veterinary doctor. [Location removed] is large and where you may desire him you will not be able to reach him. You can call the doctor about a particular situation, but he might be in Accra. And your situation may be urgent”* (Participant 6). Accessibility issues regarding animal health infrastructures were also highlighted, and several respondents indicated that more government support was needed to support biosecurity, particularly laboratory facilities for disease diagnosis, “*We need a laboratory. You can send samples and verify the cause of the disease but because we do not have a laboratory it is a big challenge”* (Participant 5).

Participants often mentioned having issues time- and labor-intensive tasks, such as cleaning and disinfecting equipment, changing the litter and refurbishing the footbath: “*Removing the drinkers and washing it is difficult because of how complicated the whole thing [cage system] is arranged.”* (Participant 3). “*Replacing water that has dried up and disinfecting the footbath is difficult. I do not forget to do it, but it is tedious. The water leaks on its own.” (FGD 2)*.

Other study participants expressed concerns about the practicality of certain biosecurity measures within the local context “*We cannot compare the foreign ways of doing things to the local setting”* (Participant 6). Factors such as climate, especially related to personal protective equipment were highlighted “*I used to wear a [gum boots] but there is so much heat in it. So, I wear a rubber sandal mostly worn by hospital workers”* (Participant 4).

Similarly, general discomfort from some biosecurity measures unrelated to the local context, such as skin irritation from disinfectant or dust when cleaning, was reported. “*What is difficult is the removal of cobwebs because even when you wear a nose mask you still feel the dust.”* (Participant 12).

Economic constraints were mentioned by a majority of participants. Participants highlighted a widespread practice in Ghana regarding withdrawal periods for drugs used in poultry farming (i.e., the period in which poultry products should not be consumed after administration with antimicrobials). The costs of adhering to these withdrawal periods were perceived as too high. Indeed, some participants mentioned that while drugs often have specified withdrawal periods, it is the customary practice not to adhere to these guidelines. For example, one participant stated, “*Most of the drugs have withdrawal periods written on them, for instance, 5 days, but in Ghana here, it is difficult to say you will give your bird feed and, after laying, wait for 5 days before releasing the eggs; you will definitely sell it.”* (Participant 3).

##### 3.2.2.2 TDF: social influences (those interpersonal processes that can cause individuals to change their thoughts, feelings, or behaviors)

Participants gauged the appropriateness of biosecurity not in absolute terms but relative to other participants in their area. One participant shared, “*My [pen] is a bit dirty but I have seen worse.”*

Additionally, some participants mentioned feeling external pressure to both adhere to certain biosecurity measures and engage in practices contrary to accepted biosecurity. For the former, in FGD 1, a participant noted “*When you enter the farm, you can detect the odor [of diseased birds] so it is mandatory to dispose of the dead birds to prevent odor most especially when living in a developed area.”* (FGD 1). In terms of the latter, one participant responded, when asked about the disposal of dead animals “*At first I used to have a garden I bury them in but the truth is that some people saw it and they asked that I give the dead animals to them so that is what I do”* (Participant 7).

Some participants receive support from different sources, including their family household and management “*I have delegated the cleaning of the drinkers to the children”* (Participant 7). Another participant expressed satisfaction with the support they generally receive, stating that their ideas are well-received and supported by their management “*I am very happy, because it is a [removed to ensure confidentiality] whatever idea I bring they buy into it”*. (Participant 10). Participants expressed a desire for more support from the government and believed that government policies do not adequately address their specific needs. One participant further said that they are losing interest in farming due to the lack of government support: “*The government will rather give loans to [businesses] ignoring poultry farmers so we are relying on our own strength. As I speak now, I am no longer interested in farming because a lot has changed in 1 year.”* (Participant 6).

#### 3.2.3 Motivation

Motivation reflects the brain processes which direct behavior. Motivation can be automatic, for instance, based on emotions or impulses, or it can be reflective because of evaluations and plans.

##### 3.2.3.1 TDF: professional role and identity (a coherent set of behaviors and displayed personal qualities of an individual in a social or work setting)

Some participants in the sample explained that they see the implementation of biosecurity as their professional duty and part of their day-to-day work, therefore, barriers such as time constraints or intensity of labor do not factor into a significant extent. This perspective is illustrated by one participant who stated, “*Because it is my job and [I] am supposed to practice it. Even if it is difficult you would have to do it*.” (Participant 1). “*I have love for the job, and I want to know all about poultry farming so that motivates me to work*.” (Participant 10).

##### 3.2.3.2 TDF: beliefs about capabilities (acceptance of the truth, reality, or validity about an ability, talent, or facility that a person can put to constructive use)

A potential lack of self-efficacy was mentioned through the belief that disease is inevitable and that it is determined by God's will. They expressed this perspective by stating, “*The animals will fall sick at any cost that is the work of God's hands and in God's plan*” (Participant 1).

##### 3.2.3.3 TDF: belief about consequences (acceptance of the truth, reality, or validity about outcomes of a behavior in each situation)

Participants also seemed to underestimate the consequences of their behavior, especially if they considered the behavior as small or insignificant. “*Sometimes we assume we will come out of the pen quickly so there is no need to change.”* (FGD 1). “*We used to think that nothing will happen even if we do not adhere.”* (FGD 2).

Proof of concept was identified as a theme through the inductive analysis. When participants saw that biosecurity is effective in reducing disease and improving productivity, they were more motivated to continue implementing it. “*[Implementing biosecurity] will enable the animals to lay more eggs.”* (Participant 2). “*If you work and see improvement that motivates you and you become happy.”* (Participant 1).

The perceived cost-benefit of biosecurity measures was identified as another theme in the inductive analysis. Several respondents expressed concerns about the financial constraints associated with biosecurity measures that demotivate them to continuously use it. One participant in the FGD summarized, “*All these practices come at a cost. I was buying disinfectant, but I realized it was adding to my cost. We are therefore trying to maximize profit in our small way, and we tend to ignore all these things.”* (FGD 2). Another participant mentioned that some producers still prefer using antimicrobials for disease prevention as they seem to get more or additional value from this “*So, for most of us, using myself as an example, I will reduce the use of the disinfectant so that I buy the antibiotic because it also boosts the immune system of the animals and increases production. I rely more on the antibiotic than the disinfectant because the disinfectant only plays the role of prevention, but we do not cast our minds on prevention. Some of us do not focus on prevention but just on the wellbeing of the birds.”* (FGD 2).

At the same time, the lack of access to drugs and the high associated costs with buying drugs functioned as critical motivator for participants to implement biosecurity to reduce their dependence on drugs. “*The drugs are expensive; one kilo of drug can cost 600 gh and if you have about 1,000 birds it can't last them for 5 days […]. Once [the drug] is available people rush for it and if you are not first you will not get some. Everything is scarce. The good drugs are not available*” (Participant 6).

## 4 Discussion

This study explored producers' biosecurity adoption and implementation behavior on smallholder layer farms in rural Ghana, using the TDF and COM-B model. Numerous factors were found to influence producers' biosecurity adoption. Participants in this study demonstrated both awareness of AMR and recommended biosecurity practices. They clearly understood the rationale behind following certain practices and the associated benefits. This aligns with results from previous work showing increased knowledge through participation in the FFS (21). However, interview data demonstrate that producers were not implementing all the biosecurity and prudent antimicrobial use practices learned through FFS participation. Furthermore, when some measures were implemented, participants often did so knowingly inadequately. This shows that knowledge and awareness alone may be necessary conditions but insufficient to drive behavior change.

Financial, economic constraints and cost-benefit considerations were named as one of the reasons preventing biosecurity, an observation consistent with studies within the poultry sector (38, 39) and other livestock sectors both in high- and low- and middle-income contexts (40–43). Several of these biosecurity measures require high initial investment (e.g., constructing a fence around the farm), which may limit producers' ability to implement all necessary biosecurity. These costs may be perceived as particularly prohibitive by producers who lease the farm premises, which may hinder their ability and willingness to invest in redesigning aspects of the farm layout. Given the leasing of poultry houses is common in Ghana, more research is needed to understand how biosecurity may be impacted by whether a farmer owns or leases housing and equipment. In addition, some participants used the cost of antimicrobials as a threshold to determine whether investing in a particular biosecurity is economically viable. If the costs of executing a specific biosecurity are believed to exceed the expenses associated with antibiotics, some participants seemed to be reluctant to implement them. This logic is consistent with descriptions of antibiotics as “quick fixes” to resolve failures in hygiene, sanitation and biosecurity (44, 45).

However, the qualitative analysis also revealed responses that diverged from discussions purely about perceived cost benefits. This has been observed in other studies as well, for instance where producers did not adapt practices that are cheaper and easier to use (46). In this sample, participants expressed concerns about the practicality of some recommended biosecurity practices within their local context. As with economic constraints, the need for feasible biosecurity solutions that align with the unique needs of smallholder producers in low- and middle-income setting has been recommended in previous studies (22, 38, 39, 47). Indeed, some producers in our study tried to simplify the process of boot cleaning by sprinkling disinfectant on the boots rather than stepping into a footbath. This adaptation, however, may have minimal positive effects on disease prevention as shoes have to be immersed in the footbath for proper disinfection. In addition, this example of a local biosecurity adaptation highlights the need for feedback from animal health professionals in rendering biosecurity measures more practical.

Participants also frequently highlighted practices they found “tedious”, particularly those requiring frequent and regular attention, such as litter management, cleaning drinkers and feeders, and maintaining the footbath. They expressed difficulties dedicating the necessary time and labor to these tasks as they often juggle various farm responsibilities and may find the labor physically taxing without assistance. At the same time, they lacked the financial means to hire experienced poultry workers and access to affordable and labor-saving technologies that would automate and streamline these processes, such as automated feeders, drinkers and disinfection systems. A result of these combined influences is that participants were likely to employ untrained personnel, involved their family members to assist with these tasks or neglect the practice or implement it (knowingly) insufficiently. This challenge is multifaceted and can be seen as both an “opportunity” barrier, as it relates to the availability of resources and labor-saving technologies, and a “capability” barrier, as it can lead to a taxed (cognitive) bandwidth—a state where individuals are overwhelmed by the demands on their cognitive resources, potentially resulting in biases and mental shortcuts (48, 49).

For instance, one cognitive bias that emerged from the data was *temporal discounting*. Temporal discounting refers to the tendency to assign a higher value to immediate rewards or losses compared to those occurring in the future (50). Temporal discounting has been helpful in explaining behavior when there are future financial, health or environmental risks/benefits explaining unhealthy behavior such as addiction to alcohol and tobacco (51, 52), ecological outcomes such as air and water quality (53), and health outcomes such as obesity (54). In this study, we observed instances where producers prioritized avoiding small, immediate costs associated with regularly purchasing disinfectant, even though they were fully aware that the expenses for treating their chicken in the event of a disease outbreak would far exceed the cost of the disinfectant.

The interview data also indicate that producers make *probabilistic errors*, another cognitive bias. Participants in the study often admitted to occasionally neglecting the use of the footbath or wearing protective overalls when entering the poultry house, even though they were aware of their importance. This behavior suggests that producers may underestimate the significance of small probabilities. Underweighting of small probabilities may occur in a few situations: when people focus solely on the probabilities (instead of the interaction of the probabilities and potential outcomes) and the level of probability does not exceed the threshold level; when people do not have enough information to understand the probability and do not have a reference point to compare an unfamiliar risky situation to one which is well known; and when the cost of obtaining rational information about the probabilities is perceived as too high and people give up acquiring information (55).

Many producers identified strongly with their profession, which can act as an enabler as they see biosecurity as part of their professional duty and are motivated to implement it as part of good farming practices. At the same time, participants focused on maximum production and profit, which may indicate that their self-concepts are still dominated by production-oriented identities rather than identities that emphasize societal responsibility, sustainability, and a driver for change in the agricultural sector at large. These findings are consistent with previous research by other authors (56, 57), who found that producers' self-concepts are often shaped by their production-oriented identities. This can make it difficult for producers to adopt new practices, such as biosecurity, which may be seen as conflicting with their production goals.

### 4.1 Future studies and recommendations

Our results suggest several recommendations for future research and interventions to target biosecurity. First, our finding that training and awareness-raising efforts, while essential, may not be sufficient on their own suggests that these efforts should target the COM-B spectrum in parallel and include all stakeholders that are involved in the poultry business (e.g., family members and animal health professionals). This integrated approach can lead to more effective changes in biosecurity and antimicrobial use on farms, ultimately mitigating the emergence and spread of AMR. Importantly, our results show that for sustainable biosecurity interventions to be achieved, they must not only be theoretically sound but also be perceived as feasible and practical for producers in their local context. To achieve this, a bottom-up approach that involves collaboration with producers, such as the Farmer Field School approach, is crucial. Experts can facilitate this process, ensuring the effectiveness and adherence to biosecurity best practices, while producers ensure that interventions are contextualized and aligned with local circumstances and needs.

Second, fostering interdisciplinary collaboration and involving researchers from the social and behavioral sciences departments is imperative as more engagement of psychologists and other social scientists can result in the utilization of adequate theories within the veterinary sciences. Although the TDF and COM-B provide a user-friendly theoretical framework, it remains advisable to include researchers well-versed in the foundational theories throughout the research process. It might address the reason why factors related to “Motivation” and other psychological aspects of “Capability” within COM-B are less frequently identified (58). Crucially, understanding the diverse motivational triggers for livestock producers is pivotal. This understanding can serve as a valuable foundation for tailoring incentives encouraging and sustaining engagement in more pro-biosecurity behaviors.

An example of this can be seen in our categorization of the TDF domain “Beliefs About Consequences” under “Automatic Motivation” within the COM-B framework, diverging from the original suggestions by Cane et al. While the mapping might divert, it is based on a consideration of the underlying cognitive processes involved, such as cognitive biases (e.g., probabilistic errors), which can be automatic in nature (59–61). Even though participants may rationalize these processes, they still align more closely with automatic cognitive functions. Similarly, other cognitive biases identified, such as temporal discounting, might be best placed within the TDF domain “Memory, Attention, and Decision Making”. Still, they could also be considered factors influencing “Automatic Motivation” within the COM-B framework. These deliberations are significant because the COM-B framework suggests different intervention strategies for the different components (Capability, Opportunity, and Motivation), and precise categorization is crucial for designing effective interventions tailored to the specific behavioral mechanisms.

In cases where interdisciplinary research is not possible, we recommend combining the TDF and COM-B frameworks when coding qualitative interview data, as this may be a more effective approach for non-behavioral scientists than using just the broad domains of the COM-B. Similarly, veterinary medicine curricula may not give as much attention to social science aspects as they do to the biological and medical aspects of animal care. While these are essential, they are not enough for a comprehensive understanding of the complex interactions between humans, animals, and the environment. These interactions often involve behavioral, sociocultural, and economic factors that are best uncovered and understood through social and behavioral science theories and methods. As a result, veterinary professionals who are not exposed to social science disciplines such as psychology, sociology, anthropology, and economics may not be aware of the theoretical frameworks they need to generate data on underlying factors that influence behavior and decision-making.

Finally, discussing the appropriateness of psychological theoretical frameworks extends well beyond the realms of intellectual and academic curiosity; it has a profound impact on how we perceive both producers themselves and the potential behavior change interventions we may consider employing. In fact, most agricultural models dealing with producers' behavior persist in portraying them as simplistic decision-makers who uncritically implement calculated solutions (62). Recognizing the inadequacy of such assumptions becomes crucial when designing effective interventions to promote behavioral change. The COM-B and TDF frameworks offer valuable tools not only for behavioral science practitioners and non-behavioral scientists but also for researchers from the social and psychological sciences as they provide an accessible and comprehensive framework for exploring the factors influencing behavior, which can generate research questions and hypotheses. This is particularly relevant when conducting research in social-cultural contexts where the quantitative social sciences, such as psychology and behavioral economics, have yet to understand which of their (psychological) constructs can be considered universal.

### 4.2 Limitations and delimitations

Several limitations exist that limit the applicability of our results to other small-scale layer producers. First, respondents for both IDIs and FGDs were graduates of the FFS who participated in a months-long intervention that emphasized best production practices, including biosecurity and prudent antimicrobial use. While this characteristic of the study group allowed us to assess the knowledge–action gap better, as knowledge deficits were unlikely to be the reason behind poor biosecurity, it also ensured that the sample was not representative of the “typical” small-scale layer producer in Ghana. Moreover, our sampling method may have introduced a self-selection bias, as producers who willingly chose to participate in the FFS may have been more inclined to embrace the recommended production practices, which may mean that certain motivational barriers were missed.

This study's primary objective was to gain insights into smallholder poultry producers' perceptions regarding biosecurity routines and their experienced barriers and facilitators in implementing them. Consequently, the study did not aim to generalize any findings quantitatively but to uncover the factors influencing biosecurity adoption beyond knowledge and awareness. As a result, it is not feasible to determine which factors have the most significant impact on adherence to biosecurity among producers in Ghana. Furthermore, the study does not enable the drawing of causal inferences from the collected data. Second, only one woman provided response. Therefore, it is possible certain gender-related dimensions impact enablers and barriers to adoption (e.g., additional household responsibilities that further taxed cognitive bandwidth). Although the male-biased sample reflects the realities of layer production in the study area, females within the household often provide labor within the poultry house. Consequently, identifying these gender dimensions is essential in developing contextualized intervention strategies. Finally, it is crucial to recognize the affiliation of this study with a broader FAO program from which participants benefited. While participants might not have directly felt obligated to give certain answers, it cannot be excluded that some have shown a certain level of social desirability bias. Therefore, participants may have consciously or unconsciously responded in a way that they believed to be advantageous to the research program or align with the researcher's expectations (63). Consequently, the observed adherence to good biosecurity practices, as deduced solely from interviews and FGDs, might be prone to overestimation. To mitigate the impact of such biases during data analysis, it is advisable to employ triangulation by incorporating additional data sources, such as observational methods and collection of biological markers of biosecurity.

## 5 Conclusion

Thematic and framework analysis of qualitative interviews from small-scale layer producers in Ghana demonstrated that a range of factors related to capability, opportunity, and motivation impact the adoption and maintenance of biosecurity practices. Despite receiving information and training on recommended biosecurity measures during the FFS, most respondents either fail to implement all measures or do so inadequately. We found that barriers to adoption included high initial investment costs with some biosecurity measures (e.g., cleaning of drinkers/feeders) perceived as time and labor-intensive, potentially taxing their cognitive bandwidth. This, in turn, can lead to decision-making based on heuristics and cognitive biases, such as time discounting or probabilistic errors. Future research to understand on-farm biosecurity practices should embrace appropriate psychological theoretical frameworks and work in close collaboration with social and behavioral scientists. Such collaborations will be necessary to identify the enablers and barriers to patterning biosecurity practices on farms. Identifying these barriers and enablers is critical to inform the design of effective behavioral change interventions to limit health and production risks associated with disease and AMR and increase on-farm incomes.

## Data availability statement

The raw data supporting the conclusions of this article will be made available by the authors, without undue reservation.

## Ethics statement

The studies involving humans were approved by Ministry of Health Ethical Review Board (ID No. 014/10/19). The studies were conducted in accordance with the local legislation and institutional requirements. The participants provided their written informed consent to participate in this study.

## Author contributions

AB: Writing – original draft, Conceptualization, Data curation, Formal analysis, Investigation, Methodology, Project administration, Supervision. KA: Writing – review & editing, Investigation, Project administration, Resources. EKo: Writing – review & editing, Data curation, Investigation, Supervision. EKa: Funding acquisition, Resources, Writing – review & editing. CP: Conceptualization, Writing – review & editing. MC: Formal analysis, Methodology, Project administration, Writing – original draft.

## References

[1] Murray CJ Ikuta KS Sharara F Swetschinski L Robles Aguilar G Gray A. Global burden of bacterial antimicrobial resistance in 2019: a systematic analysis. Lancet. (2022) 399:629–55. 10.1016/S0140-6736(21)02724-0 35065702 PMC8841637

[2] World Bank. Pulling Together to Beat Superbugs: Knowledge and Implementation Gaps in Addressing Antimicrobial Resistance. Washington D.C.: World Bank. (2019).

[3] Martin MJ Thottathil SE Newman TB. Antibiotics overuse in animal agriculture: a call to action for health care providers. Am J Public Health. (2015) 105:2409–10. 10.2105/AJPH.2015.302870 26469675 PMC4638249

[4] FAO. The FAO Action Plan on Antimicrobial Resistance 2021-2025. Rome: Food and Agriculture Organization. (2021).

[5] Barbosa TM Levy SB. The impact of antibiotic use on resistance development and persistence. Drug Resist Updat. (2000) 3:303–11. 10.1054/drup.2000.0167 11498398

[6] European Union. Regulation (EU) 2019/6 of the European Parliament and of the Council of 11 December 2018 on Veterinary Medicinal Products and Repealing Directive 2001/82/EC. Luxembourg: Official Journal of the European Union (2018).

[7] Cox JA Vlieghe E Mendelson M Wertheim H Ndegwa L Villegas MV. Antibiotic stewardship in low- and middle-income countries: the same but different? Clin. Microbiol Infect. (2017) 23:812–8. 10.1016/j.cmi.2017.07.010 28712667

[8] Caudell MA Dorado-Garcia A Eckford S Creese C Byarugaba DK Afakye K. Towards a bottom-up understanding of antimicrobial use and resistance on the farm: a knowledge, attitudes, and practices survey across livestock systems in five African countries. PLoS ONE. (2020) 15:e0220274. 10.1371/journal.pone.0220274 31978098 PMC6980545

[9] Lowder SK Skoet J Raney T. The number, size, and distribution of farms, smallholder farms, and family farms worldwide. World Dev. (2016) 87:16–29. 10.1016/j.worlddev.2015.10.041

[10] Jimenez CEP Keestra S Tandon P Cumming O Pickering AJ Moodley A. Biosecurity and water, sanitation, and hygiene (WASH) interventions in animal agricultural settings for reducing infection burden, antibiotic use, and antibiotic resistance: a One Health systematic review. Lancet Planet Health. (2023) 7:e418–34. 10.1016/S2542-5196(23)00049-9 37164518

[11] Postma M Backhans A Collineau L Loesken S Sjölund M Belloc C. Evaluation of the relationship between the biosecurity status, production parameters, herd characteristics and antimicrobial usage in farrow-to-finish pig production in four EU countries. Porc Health Manag. (2016) 2:9. 10.1186/s40813-016-0028-z 28405435 PMC5382489

[12] Renault V Damiaans B Sarrazin S Humblet M-F Dewulf J Saegerman C. Biosecurity practices in Belgian cattle farming: level of implementation, constraints and weaknesses. Transbound Emerg Dis. (2018) 65:1246–61. 10.1111/tbed.12865 29566303

[13] Wong JT de Bruyn J Bagnol B Grieve H Li M Pym R. Small-scale poultry and food security in resource-poor settings: a review. Glob Food Secur. (2017) 15:43–52. 10.1016/j.gfs.2017.04.003

[14] Wauters E Rojo-Gimeno C. Socio-Psychological Veterinary Epidemiology: A New Discipline for an Old Problem? (2014). Available online at: https://www.researchgate.net/publication/261438047_Socio-psychological_veterinary_epidemiology_a_new_discipline_for_an_old_problem (accessed September 18, 2023).

[15] Biesheuvel MM Santman-Berends IMGA Barkema HW Ritter C Berezowski J Guelbenzu M. Understanding farmers' behavior and their decision-making process in the context of cattle diseases: a review of theories and approaches. Front Vet Sci. (2021) 8. 10.3389/fvets.2021.687699 34926632 PMC8674677

[16] Burton RJF. Reconceptualising the ‘behavioural approach' in agricultural studies: a socio-psychological perspective. J Rural Stud. (2004) 20:359–71. 10.1016/j.jrurstud.2003.12.001

[17] Afakye K Kiambi S Koka E Kabali E Dorado-Garcia A Amoah A. The impacts of animal health service providers on antimicrobial use attitudes and practices: an examination of poultry layer farmers in Ghana and Kenya. Antibiotics. (2020) 9:554. 10.3390/antibiotics9090554 32872381 PMC7557566

[18] Garforth CJ Bailey AP Tranter RB. Farmers' attitudes to disease risk management in England: a comparative analysis of sheep and pig farmers. Prev Vet Med. (2013) 110:456–66. 10.1016/j.prevetmed.2013.02.018 23490144

[19] Pham-Duc P Cook MA Cong-Hong H Nguyen-Thuy H Padungtod P Nguyen-Thi H. Knowledge, attitudes and practices of livestock and aquaculture producers regarding antimicrobial use and resistance in Vietnam. PLoS ONE. (2019) 14:e0223115. 10.1371/journal.pone.0223115 31553776 PMC6760827

[20] Mangesho PE Caudell MA Mwakapeje ER Ole-Neselle M Kimani T Dorado-García A. Knowing is not enough: a mixed-methods study of antimicrobial resistance knowledge, attitudes, and practises among maasai pastoralists. Front Vet Sci. (2021) 8. 10.3389/fvets.2021.645851 33834048 PMC8023390

[21] Caudell M Mangesho PE Mwakapeje ER Dorado-García A Kabali E Price C. Narratives of veterinary drug use in northern Tanzania and consequences for drug stewardship strategies in low-income and middle-income countries. BMJ Glob Health. (2022) 7:e006958. 10.1136/bmjgh-2021-006958 35058305 PMC8772431

[22] Pao H Jackson E Yang T Tsai J Sung WHT Pfeiffer DU. Determinants of farmers' biosecurity mindset: A social-ecological model using systems thinking. Front Vet Sci. (2022) 9. 10.3389/fvets.2022.959934 36046509 PMC9420990

[23] Davidson D. Radical interpretation. Dialectica. (1973) 1973:313–328. 10.1111/j.1746-8361.1973.tb00623.x

[24] Heider F. The Psychology of Interpersonal Relations. London: Psychology Press. (2013). 10.4324/9780203781159

[25] Sellars W. Empiricism and the philosophy of mind. Minn Stud Philos Sci. (1956) 1:253–329.

[26] Elster J. Rational Choice. New York: NYU Press. (1986).

[27] Michie S van Stralen MM West R. The behaviour change wheel: a new method for characterising and designing behaviour change interventions. Implement Sci. (2011) 6:42. 10.1186/1748-5908-6-42 21513547 PMC3096582

[28] Cane J O'Connor D Michie S. Validation of the theoretical domains framework for use in behaviour change and implementation research. Implement Sci. (2012) 7:37. 10.1186/1748-5908-7-37 22530986 PMC3483008

[29] Michie S Johnston M Abraham C Lawton R Parker D Walker A. Making psychological theory useful for implementing evidence based practice: a consensus approach. Qual Saf Health Care. (2005) 14:26–33. 10.1136/qshc.2004.011155 15692000 PMC1743963

[30] Farrell S Benson T McKernan C Regan Á Burrell AMG Dean M. Factors influencing dairy farmers' antibiotic use: An application of the COM-B model. J Dairy Sci. (2023) 106:4059–71. 10.3168/jds.2022-22263 37028957

[31] Farrell S Benson T McKernan C Regan Á Burrell AMG Dean M. Exploring veterinarians' behaviour relating to antibiotic use stewardship on Irish dairy farms using the COM-B model of behaviour change. Res Vet Sci. (2023) 156:45–53. 10.1016/j.rvsc.2023.01.019 36780797

[32] Furtado T Perkins E Pinchbeck G McGowan C Watkins F Christley R. Exploring human behavior change in equine welfare: insights from a COM-B analysis of the UK's equine obesity epidemic. Front Vet Sci. (2022) 9:961537. 10.3389/fvets.2022.961537 36425120 PMC9681534

[33] Millar N., Dufour, S., Lard,é, H., Roy, J.-P., Belloc, C., Francoz, D., et al. (2023). Barriers and facilitators to implementing a new regulation restricting antimicrobial use in dairy production in Québec, Canada: a qualitative study. Front Vet Sci. 10:1025781. 10.3389/fvets.2023.1025781 37008362 PMC10060835

[34] Caudell MA Kiambi S Afakye K Koka E Kabali E Kimani T. Social-technical interventions to reduce antimicrobial resistance in agriculture: evidence from poultry Farmer Field Schools in Ghana and Kenya. JAC-Antimicrob Resist. (2022) 4:dlab193. 10.1093/jacamr/dlab193 35156026 PMC8826779

[35] FAO. Poultry Sector Ghana. FAO Animal Production and Health Livestock Country Reviews No. 6. Rome: FAO (2014).

[36] Rampin R Rampin V. Taguette: open-source qualitative data analysis. J Open Source Softw. (2021) 6:3522. 10.21105/joss.03522

[37] Braun V Clarke V. Using thematic analysis in psychology. Qual Res Psychol. (2006) 3:77–101. 10.1191/1478088706qp063oa32100154

[38] Conan A Goutard FL Sorn S Vong S. Biosecurity measures for backyard poultry in developing countries: a systematic review. BMC Vet Res. (2012) 8:1–10. 10.1186/1746-6148-8-240 23216706 PMC3538710

[39] Rimi N Sultana R Muhsina M Uddin B Haider N Nahar N. Biosecurity conditions in small commercial chicken farms, Bangladesh 2011–2012. Ecohealth. (2017) 14:244–58. 10.1007/s10393-017-1224-2 28289988 PMC5942227

[40] Dione M Ouma E Opio F Kawuma B Pezo D. Qualitative analysis of the risks and practices associated with the spread of African swine fever within the smallholder pig value chains in Uganda. Prev Vet Med. (2016) 135:102–12. 10.1016/j.prevetmed.2016.11.001 27931922

[41] Ebata A MacGregor H Loevinsohn M Win KS. Why behaviours do not change: structural constraints that influence household decisions to control pig diseases in Myanmar. Prev Vet Med. (2020) 183:105138. 10.1016/j.prevetmed.2020.105138 32977171

[42] Fraser RW Williams N Powell L Cook A. Reducing Campylobacter and salmonella infection: two studies of the economic cost and attitude to adoption of on-farm biosecurity measures. Zoonoses Public Health. (2010) 57:e109–15. 10.1111/j.1863-2378.2009.01295.x 19968845

[43] Msimang V Rostal MK Cordel C Machalaba C Tempia S Bagge W. Factors affecting the use of biosecurity measures for the protection of ruminant livestock and farm workers against infectious diseases in central South Africa. Transbound Emerg Dis. (2022) 69:e1899–912. 10.1111/tbed.14525 35306739 PMC9790579

[44] Chandler CI. Current accounts of antimicrobial resistance: stabilisation, individualisation and antibiotics as infrastructure. Palgrave Commun. (2019) 5:1–13. 10.1057/s41599-019-0263-4 31157116 PMC6542671

[45] Willis LD Chandler C. Quick fix for care, productivity, hygiene and inequality: reframing the entrenched problem of antibiotic overuse. BMJ Glob Health. (2019) 4:e001590. 10.1136/bmjgh-2019-001590 31497315 PMC6703303

[46] Can MF Altug N. Socioeconomic implications of biosecurity practices in small-scale dairy farms. Vet Q. (2014) 34:67–73. 10.1080/01652176.2014.951130 25174643

[47] Wolff C Abigaba S Sternberg Lewerin S. Ugandan cattle farmers' perceived needs of disease prevention and strategies to improve biosecurity. BMC Vet Res. (2019) 15:208. 10.1186/s12917-019-1961-2 31226988 PMC6588948

[48] Mani A Mullainathan S Shafir E Zhao J. Poverty impedes cognitive function. Science. (2013) 341:976–80. 10.1126/science.1238041 23990553

[49] Schilbach F Schofield H Mullainathan S. The psychological lives of the poor. Am Econ Rev. (2016) 106:435–40. 10.1257/aer.p20161101 29547249

[50] Samson A editor. The Behavioral Economics Guide 2017 (With an Introduction by Cass Sunstein) (2017). Available online at: http://www.behavioraleconomics.com (accessed September 19, 2023).

[51] Roewer I Wiehler A Peters J. Nicotine deprivation, temporal discounting and choice consistency in heavy smokers. J Exp Anal Behav. (2015) 103:62–76. 10.1002/jeab.134 25641080

[52] Scharff RL Viscusi WK. Heterogeneous rates of time preference and the decision to smoke. Econ Inq. (2011) 49:959–72. 10.1111/j.1465-7295.2009.00191.x 22165417

[53] Hardisty DJ Weber EU. Discounting future green: money versus the environment. J Exp Psychol Gen. (2009) 138:329. 10.1037/a0016433 19653793

[54] Richards TJ Hamilton SF. Obesity and hyperbolic discounting: an experimental analysis. J Agric Resour Econ. (2012) 181–98.

[55] Tyszka T Zielonka P. Overweighting versus underweighting of small probabilities. In: Large Risks with Low Probabilities: Perceptions and Willingness to Take Preventive Measures Against Flooding. London: IWA Publishing. (2017) p. 41–58.

[56] Burton RJF Wilson GA. Injecting social psychology theory into conceptualisations of agricultural agency: towards a post-productivist farmer self-identity? J Rural Stud. (2006) 22:95–115. 10.1016/j.jrurstud.2005.07.004

[57] Burton RJ. Seeing through the ‘good farmer's' eyes: towards developing an understanding of the social symbolic value of ‘productivist'behaviour. Sociol Rural. (2004) 44:195–215. 10.1111/j.1467-9523.2004.00270.x

[58] Mather M Pettigrew LM Navaratnam S. Barriers and facilitators to clinical behaviour change by primary care practitioners: a theory-informed systematic review of reviews using the Theoretical Domains Framework and Behaviour Change Wheel. Syst Rev. (2022) 11:180. 10.1186/s13643-022-02030-2 36042457 PMC9429279

[59] Kahneman D Tversky A. Subjective probability: a judgment of representativeness. Cognit Psychol. (1972) 3:430–54. 10.1016/0010-0285(72)90016-3

[60] Tversky A Kahneman D. Judgment under uncertainty: heuristics and biases: biases in judgments reveal some heuristics of thinking under uncertainty. Science. (1974) 185:1124–31. 10.1126/science.185.4157.1124 17835457

[61] Tversky A Kahneman D. Availability: a heuristic for judging frequency and probability. Cognit Psychol. (1973) 5:207–32. 10.1016/0010-0285(73)90033-9

[62] Findlater K Satterfield T Kandlikar M. Farmers' risk-based decision making under pervasive uncertainty: cognitive thresholds and hazy hedging. Risk Anal. (2019). 10.1111/risa.13290 30830976

[63] Kaminska O Foulsham T. Understanding sources of social desirability bias in different modes: evidence from eye-tracking. In: ISER Working Paper Series. Colchester: University of Essex; Institute for Social and Economic Research (ISER) (2013).

